# Wandering Liver: A Case Report With Clinical and Radiological Insights

**DOI:** 10.7759/cureus.72025

**Published:** 2024-10-21

**Authors:** Andrés Felipe Herrera Ortiz, Laura Olarte, Diego A Aguirre, Lorena F Fernández Beaujon, Sergio Castellanos, Diana Romero, Rubén Giraldo Malo, Natalia Gutiérrez Romero, Alejandro J Quiroz Alfaro

**Affiliations:** 1 Radiology Department, Fundación Santa Fe de Bogotá, Bogotá D.C., COL; 2 Radiology Department, Universidad El Bosque, Bogotá D.C., COL; 3 Medicine Department, Universidad El Bosque, Bogotá D.C., COL; 4 Medicine Department, Universidad del Rosario, Bogotá D.C., COL; 5 Internal Medicine Department, North Mississippi Medical Center, Tupelo, USA

**Keywords:** anatomical variants of the liver, falciform ligament, hepatopexy, liver infarction, wandering liver

## Abstract

Wandering liver (WL) is an exceptionally rare anatomical variant, scarcely described in the medical literature. This condition is characterized by hypermobility of the liver within the abdominal cavity, resulting from the weakening, laxity, or absence of the liver's suspensory ligaments. This case report describes a 28-year-old male patient with a history of Sashi-Pena syndrome who presented with chronic, nonspecific abdominal pain, in which cross-sectional imaging incidentally revealed WL. This case report seeks to provide an overview of WL, emphasizing crucial anatomical information that radiologists should include in their assessments.

## Introduction

The liver is essential in various physiological functions, including nutrient metabolism, protein synthesis, vitamin storage, and regulation of carbohydrate and lipid levels [1,2].

The liver is anatomically divided into eight distinct segments, each with its own independent blood supply and bile drainage. At the liver's hilum, or porta hepatis, crucial structures enter and exit, including the hepatic artery, hepatic portal vein, right and left hepatic ducts, lymphatic vessels, and autonomic nerves, all vital for its vascular supply and functional integrity [1]. The liver is anchored in the right upper quadrant of the abdomen by key ligaments, including the falciform ligament, ligamentum teres, coronary ligaments, and the right and left triangular ligaments. These structures provide stability and help define its positional relationship within the peritoneal cavity [1].

Wandering liver (WL) is a rare and poorly understood condition that occurs when the liver becomes mobile in the abdominal cavity due to a weakening, laxity, or absence of these supportive ligaments [3]. This unusual mobility allows the liver to migrate freely, typically from the right side of the abdomen to the left.

Currently, only about 25 cases of WL have been documented in the medical literature, highlighting the rarity of this condition [4]. This limited data about WL underscores the need for more cases to be reported to fully understand its clinical features, diagnostic challenges, treatment options, and long-term outcomes. Here, we present a case of a 28-year-old male patient with WL, highlighting key anatomic aspects that radiologists must include in their reports.

## Case presentation

**Figure 1:**
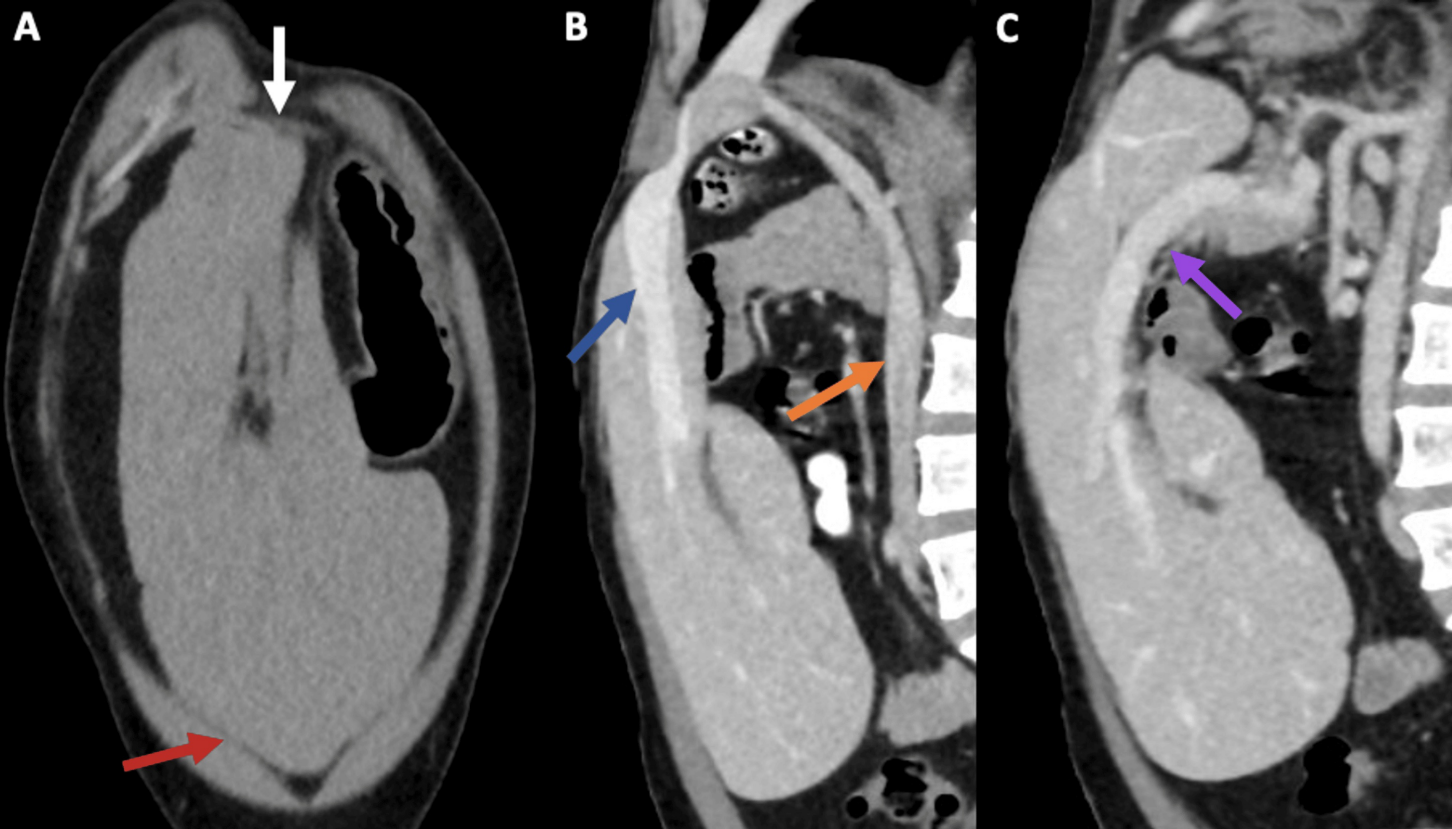
An abdominal CT scan (A) Coronal non-contrast CT scan showing a vertically orientated liver in the midline, with the right hepatic lobe located caudally (red arrow); notice that the left hepatic triangular ligament is anchoring the left hepatic lobe to the diaphragm (white arrow). (B)-(C) Sagittal contrast-enhanced CT scan in venous phase showing that the hepatic hilum is located cephalically; notice the suprahepatic veins (blue arrow), inferior vena cava (orange arrow), and the portal vein (purple arrow). CT: Computed tomography

**Figure 2:**
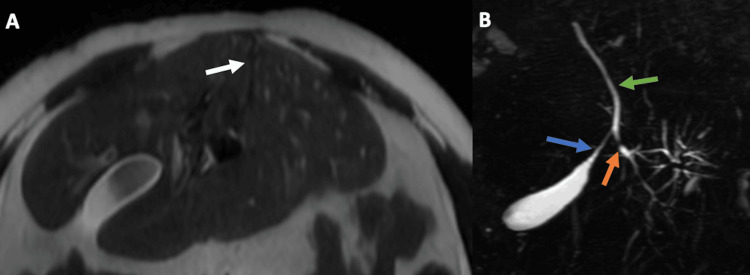
An MRCP scan (A) Axial T2 sequence showing the falciform ligament anchored to the anterior abdominal wall (white arrow). (B) Radial sequences showing the common hepatic duct (orange arrow), cystic duct (blue arrow), and common bile duct (green arrow) with a cephalic orientation toward the ampulla of Vater, which has a usual anatomical location in the second portion of the duodenum. MECP: Magnetic resonance cholangiopancreatography

A 28-year-old male patient with a history of Sashi-Pena syndrome presented at the Emergency Department with a three-month history of moderate-intensity abdominal pain localized in the epigastrium, without any other symptoms. Physical examination revealed a hardened bulge in the epigastric region. Laboratory tests showed normal blood, renal, and liver function. An abdominal computed tomography (CT) scan was ordered to rule out neoplasia, which incidentally revealed a vertically rotated liver in the midline, consistent with WL (Figure 1). To further characterize the bile system and potentially rule out surgical conditions, magnetic resonance cholangiopancreatography (MRCP) was requested, showing no choledocholithiasis (Figure 2). Analgesia with naproxen was administered to relieve the pain, which subsequently resolved. After ruling out other potential diagnoses, the pain was attributed to gastritis; therefore, the patient was discharged with an ambulatory control appointment.

## Discussion

WL is an extremely rare condition, with only 25 cases reported in the medical literature affecting adults and children [4]. WL can be both acquired and congenital, with the former being more frequent [5]. Currently, no clear syndromic associations have been established [6].

WL is typically asymptomatic, but in rare cases, it can cause intermittent abdominal pain due to the torsion and detorsion of the vascular pedicle, potentially leading to hepatic ischemia and infarction [7]. WL is often associated with laxity or a lack of the sigmoid mesocolon, predisposing patients to sigmoid colon volvulus [6]. In this case, no hepatic infarction or inflammatory changes in the hepatic parenchyma were documented on cross-sectional imaging, ruling out hepatic vascular pedicle torsion.

**Figure 3:**
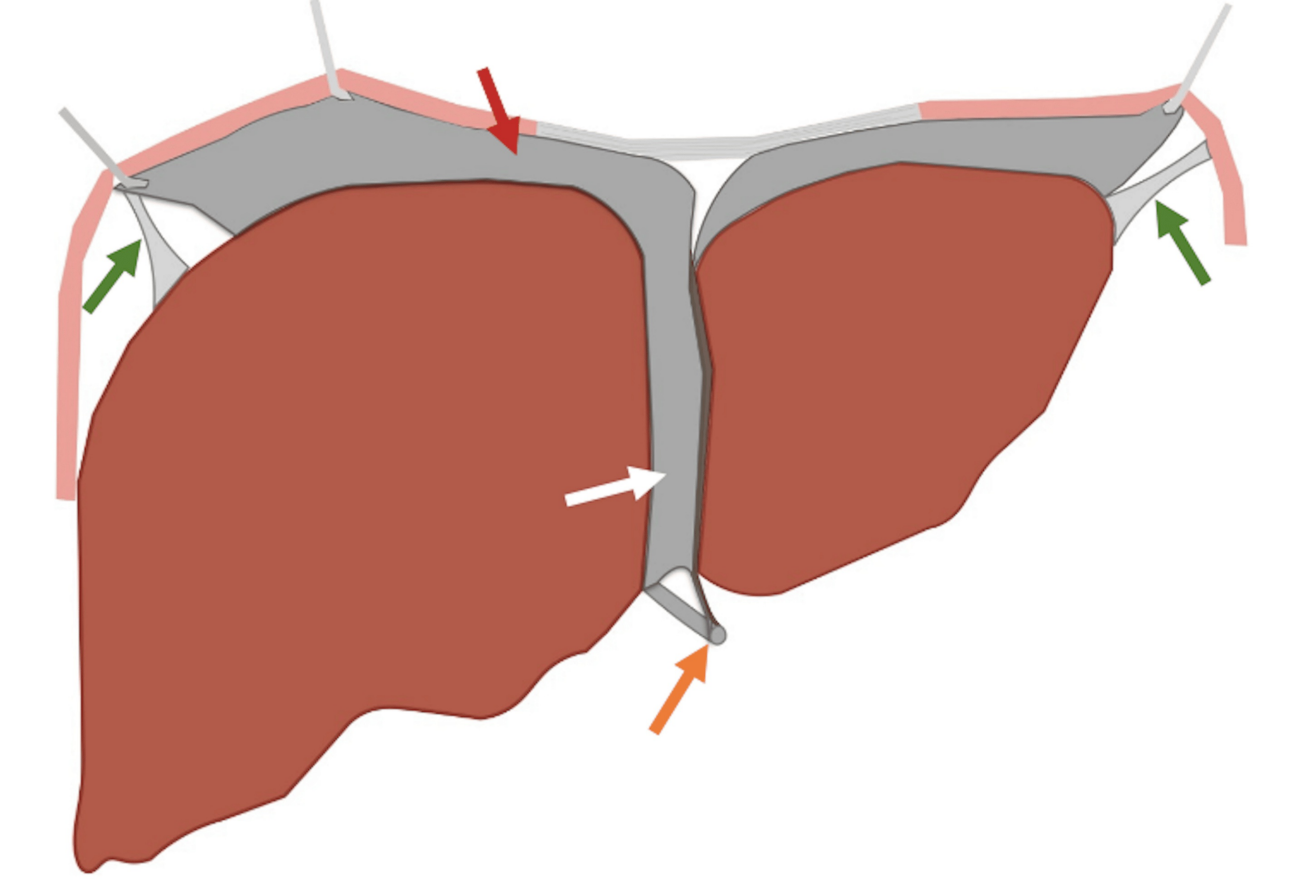
Illustration depicting the hepatic suspensory ligaments Right and left triangular ligaments (green arrows), coronary ligament (red arrow), falciform ligament (white arrow) and ligamentum teres (orange arrow). Image credit: Illustration elaborated by the authors

The definitive diagnosis of WL is achieved through cross-sectional imaging modalities such as CT and magnetic resonance imaging (MRI), which provide detailed anatomical assessments [8]. It is crucial for radiologists to accurately identify the presence of the falciform ligament, coronary ligaments, and triangular ligaments, as well as the configuration of the vascular supply and bile duct system (Figure 3). In this case, the liver was positioned in the midline, primarily due to partial anchoring by the falciform ligament to the anterior abdominal wall and the partial attachment of the left hepatic triangular ligament to the diaphragm. This resulted in a partially, but not entirely, mobile liver.

In this case, the hepatic hilum was situated cephalically, with the suprahepatic and porta veins positioned superiorly. It is crucial to document this anatomical orientation of the hepatic hilum, particularly in the context of planned hepatopexy procedures. Despite the liver’s vertical orientation in the midline, the common bile duct ascended to drain normally into the ampulla of Vater, located at the second portion of the duodenum.

The management of WL remains challenging due to its rarity and nonspecific clinical presentation. Generally, two treatment approaches are considered. Expectant management is typically recommended for asymptomatic patients or those with mild symptoms [5]. However, in cases where patients present with hepatic pedicle torsion or infarction, surgical intervention, such as laparoscopic hepatopexy, is recommended [5]. This procedure repositions and stabilizes the liver in the upper right quadrant, reducing the risk of ischemia and infarction [5]. Hepatopexy involves securing the liver to the abdominal wall using non-absorbable sutures, typically utilizing the falciform ligament as the primary anchor point [5]. Therefore, accurately determining the presence or absence of the falciform ligament is crucial, as it can significantly impact treatment decisions.

In general, the prognosis of WL is favorable, as most cases are asymptomatic [5]. However, when complications such as hepatic pedicle torsion or infarction occur, they can significantly increase morbidity. Early detection and appropriate management are essential to prevent these complications and ensure a good outcome [5].

## Conclusions

WL is a rare condition that presents distinctive diagnostic and therapeutic challenges. It is essential for radiologists to be familiar with the liver's suspensory ligaments and to report the presence or absence of the falciform ligament, as this can directly impact treatment decisions. Furthermore, recognizing potential complications of WL, such as liver infarction due to pedicle torsion, is critical for patient management. We encourage readers to contribute additional case reports to help expand the literature and deepen understanding of this uncommon condition.
